# Dietary *Scutellaria baicalensis* polysaccharide as an antibiotic alternative improves growth performance, antioxidant capacity, and intestinal mucosal immunity in broiler chickens

**DOI:** 10.14202/vetworld.2026.339-354

**Published:** 2026-01-25

**Authors:** Shouzhen Liu, Qianmei Zhang, Yunhe Wang, Wenqing Zhu, Lanxin Li, Yong Zhang, Jing Zhang

**Affiliations:** 1 College of Veterinary Medicine, Gansu Agricultural University, Lanzhou city, Gansu Province, P. R. China; 2 Key Laboratory of Animal Reproductive Physiology and Reproductive Regulation of Gansu Province, Lanzhou city, Gansu Province, P. R. China; 3 Institute of Laboratory Animal Science, Guizhou University of Traditional Chinese Medicine, Guiyang City, Guizhou Province, P. R. China

**Keywords:** antibiotic alternative, antioxidant capacity, broiler chickens, gut immunity, intestinal morphology, *Scutellaria baicalensis* polysaccharide, Traditional Chinese medicine, growth performance

## Abstract

**Background and Aim:**

The global ban on antibiotic growth promoters in poultry production has accelerated the search for safe and effective natural alternatives. Polysaccharides derived from traditional Chinese medicinal plants have shown promise due to their antioxidant and immunomodulatory properties. This study evaluated the efficacy of dietary *Scutellaria baicalensis* polysaccharide (SBP) as an alternative to in-feed antibiotics by assessing its impact on growth performance, antioxidant status, digestive function, intestinal morphology, and mucosal immunity in broiler chickens.

**Materials and Methods:**

A total of 420 one-day-old Arbor Acre broilers were randomly assigned to five dietary treatments for 42 days, with six replicates per treatment. The treatments included an antibiotic-free basal diet (control), a basal diet supplemented with colistin sulfate and virginiamycin (antibiotics), and the basal diet supplemented with SBP at 100 mg/kg (SBP-L), 200 mg/kg (SBP-M), or 400 mg/kg (SBP-H). Growth performance parameters were recorded, and on days 21 and 42, serum and intestinal antioxidant indices, digestive enzyme activities, intestinal morphology, secretory immunoglobulin A (sIgA) levels, and the expression of immune-related genes (C-C motif chemokine ligand 28 [CCL28], A proliferation-inducing ligand (tumor necrosis factor ligand superfamily member 13) [APRIL]) and toll-like receptor 4 protein were evaluated.

**Results:**

Dietary SBP supplementation significantly improved average daily gain (ADG) and feed conversion ratio during the starter phase without affecting feed intake or mortality (p < 0.05). Over the entire 42-day period, broilers fed 400 mg/kg SBP showed a 3.4% higher ADG than those receiving antibiotics (p < 0.05). SBP boosted systemic and intestinal antioxidant capacity by increasing glutathione peroxidase, glutathione reductase, Superoxide dismutase, and total antioxidant capacity activities while lowering malondialdehyde levels (p < 0.05). Additionally, SBP increased digestive enzyme activities, improved villus height-to-crypt depth ratios, and raised sIgA concentrations in the duodenum and jejunum. The upregulation of TLR4 protein and the immune-related genes CCL28 and APRIL indicated enhanced intestinal mucosal immunity, especially in the SBP-M and SBP-H groups.

**Conclusion:**

Dietary supplementation with *S. baicalensis* polysaccharide, especially at 200–400 mg/kg, effectively improves growth performance, antioxidant defense, and intestinal health in broilers, demonstrating its strong potential as a practical and sustainable alternative to antibiotic growth promoters in poultry production.

## INTRODUCTION

The nationwide ban on antibiotic growth promoters in Chinese poultry production, implemented in 2020, marks a major shift in animal farming practices and reflects a global move toward more sustainable livestock practices. This policy change has heightened the need for effective, safe, and sustainable alternatives to antibiotics, both nationally and internationally, especially as producers face common issues such as reduced growth performance and increased vulnerability to disease [1, 2]. As a result, natural feed additives are gaining increasing interest, with traditional Chinese medicine (TCM) emerging as a promising approach to support animal health and productivity [1, 2].

The importance of this research is supported by the long history and well-documented bioactivities of TCM herbs. Among them, *Scutellaria baicalensis* (Huang Qin) is a key medicinal plant widely used in traditional practice and supported by substantial modern scientific evidence of its therapeutic potential [3]. Its uses go beyond traditional applications, with reported benefits in disease prevention and health promotion across various mammalian models and clinical conditions, including diabetes-related complications [4], gastric cancer [5], and ulcerative colitis [6]. In animal production systems, various TCM formulations have produced promising results, enhancing growth performance and overall health in species such as beef cattle, pigs, broiler chickens, and lactating dairy cows [7–11].

Polysaccharides derived from medicinal herbs form a particularly promising group of bioactive compounds, increasingly recognized for their wide-ranging biological functions, including strong antioxidant, anti-inflammatory, and immunomodulatory activities [12–14]. These compounds have become a central focus of scientific research, especially in biomedical sciences, due to their complex structures and diverse mechanisms of action [15]. In poultry nutrition, accumulating evidence shows that TCM–derived polysaccharides provide multiple benefits, such as enhancing nutrient digestion and absorption [16], improving growth performance [17], positively affecting meat quality attributes [18], strengthening systemic and intestinal antioxidant defenses [17, 19], and reinforcing the intestinal barrier function [20].

Despite the increasing evidence supporting the use of TCM–derived polysaccharides as functional feed additives in poultry, several important knowledge gaps remain. Most current studies have concentrated on single outcomes, such as growth performance or specific immune markers, without offering a comprehensive assessment of systemic antioxidant status, intestinal redox balance, digestive capacity, mucosal immunity, and gut morphology within a unified experimental framework. Notably, although *S. baicalensis* is a well-known medicinal herb, research on its purified polysaccharide fraction in broiler chickens is very limited, and data comparing it with conventional in-feed antibiotics are scarce. Additionally, the dose–response effects of *S. baicalensis* polysaccharide (SBP) on broiler performance and gut health parameters have not been systematically explored, which limits its potential for practical use in commercial feed formulations.

Mechanistically, the pathways by which herbal polysaccharides enhance intestinal mucosal immunity in poultry remain poorly understood. Although polysaccharides are known to have immunomodulatory effects, their impact on key pattern recognition receptors (PRRs), such as toll-like receptor 4 (TLR4), and downstream mucosal immune mediators, including secretory immunoglobulin A (sIgA), A proliferation-inducing ligand (tumor necrosis factor ligand superfamily member 13) [APRIL], and C-C motif chemokine ligand 28 [CCL28] have not been thoroughly studied in broilers. Furthermore, the connection between enhanced antioxidant capacity, digestive enzyme activity, intestinal structure, and immune activation has rarely been examined together. As a result, there is a significant lack of comprehensive, mechanistic research directly comparing SBP with antibiotic growth promoters under standardized production conditions, especially in post-antibiotic poultry systems.

The present study was designed to systematically evaluate the potential of SBP as a natural alternative to in-feed antibiotics in broiler chickens. Specifically, the objectives were to (i) assess the dose-dependent effects of dietary SBP supplementation on growth performance and feed efficiency; (ii) determine its impact on systemic and intestinal antioxidant status; (iii) evaluate changes in digestive enzyme activities and intestinal morphology; and (iv) elucidate its role in enhancing intestinal mucosal immunity by examining sIgA production and the expression of key immune-related genes (APRIL and CCL28) and TLR4 protein. By integrating performance, biochemical, histological, and molecular analyses, this study aims to provide a comprehensive mechanistic understanding of SBP’s action and to establish an effective, commercially relevant supplementation range for sustainable, antibiotic-free broiler production.

## MATERIALS AND METHODS

### Ethical approval

The animal-related experimental protocol was reviewed and approved by the Institutional Animal Care and Use Committee (IACUC) at Guizhou University of Traditional Chinese Medicine in Guiyang, China (approval no. 20210173; approved on July 25, 2021). All procedures involving animal handling, housing, management, sampling, and euthanasia were performed strictly in accordance with IACUC guidelines and the National Institutes of Health Guide for the Care and Use of Laboratory Animals. Every effort was made to minimize animal suffering and reduce the number of animals used while maintaining the reliability and scientific validity of the results.

### Study period and location

The study was conducted from August 3, 2021, to September 30, 2021. All experimental procedures and data collection were conducted at the Institute of Laboratory Animal Science, Guizhou University of Traditional Chinese Medicine (Guiyang City, China).

### Experimental birds and housing conditions

A total of 420 one-day-old Arbor Acre broiler chicks were obtained from a local commercial hatchery (Guizhou Arbor Acres Poultry Breeding Co. Ltd., China). Throughout the experimental period, husbandry and environmental conditions were maintained according to previously published protocols [21]. The ambient room temperature was maintained at 33°C ± 1°C during the first 3 days and was gradually reduced by approximately 2°C–3°C per week until a final temperature of 22°C ± 1°C was reached by the end of the experiment. Relative humidity was maintained at 60% ± 10% throughout the trial.

A lighting program of 23 h light and 1 h darkness (23L:1D) was provided during the first 7 days, followed by 18 h light and 6 h darkness (18L:6D) from days 8 to 42, with a light intensity of approximately 20 lux at bird level. Mechanical ventilation systems ensured a continuous supply of fresh air and removed excess moisture and ammonia, maintaining ammonia concentrations below 15 ppm. Birds were housed in cages with raised wire floors, and litter material was not used in this cage-rearing system. Droppings were regularly removed to maintain hygienic conditions.

All cages were of the same size. Each replicate cage housed 14 birds, resulting in an initial stocking density of about 450 cm² per bird, which allowed sufficient space throughout the 42-day rearing period in line with animal welfare guidelines. All broilers had free access to mash feed and water supplied through trough feeders and nipple drinkers.

### Experimental design and dietary treatments

All birds were randomly assigned to five dietary treatments using a completely randomized design (CRD). Briefly, chicks were weighed at placement, and birds with similar initial body weights were grouped and then randomly allocated to one of the five dietary treatments using a random number generator, ensuring similar average starting weights among treatments. Each dietary treatment included six replicate cages with 14 birds per replicate. This experimental setup provided enough statistical power to detect biologically relevant differences in growth performance parameters.

The dietary treatments included: an antibiotic-free basal diet (control); the basal diet supplemented with 20 mg/kg colistin sulfate and 20 mg/kg virginiamycin (antibiotics); and the basal diet supplemented with 100 mg/kg SBP (SBP-L), 200 mg/kg SBP (SBP-M), or 400 mg/kg SBP (SBP-H). The selected SBP dosages were based on effective ranges reported for polysaccharides derived from other traditional Chinese medicinal herbs in broiler chickens [22–24]. A dose–response design incorporating low, medium, and high SBP levels was used to find the best effective supplementation level. No adverse effects were seen at any supplementation level, showing that all tested doses were safe for broiler diets.

Colistin sulfate (purity 10%) was obtained from Guizhou Pharmaceutical Group Co. Ltd. (Guiyang, Guizhou, China), and virginiamycin (purity 50%) was purchased from Phibro Animal Health (Teaneck, NJ, USA). *S. baicalensis* polysaccharide was supplied by Guizhou Pharmaceutical Group Co. Ltd. (Guiyang, Guizhou, China). The SBP was extracted from the roots of *S. baicalensis* Georgi using hot water extraction, followed by ethanol precipitation according to the manufacturer’s instructions. The final product was a brown powder with a total polysaccharide content exceeding 85% (w/w), as determined by the phenol–sulfuric acid method. The feeding trial lasted 42 days, and all broilers had ad libitum access to mash feed and water throughout the experiment.

### Basal diet composition and feed preparation

The composition and nutrient levels of the basal diets are shown in Table 1 and were formulated to meet the nutrient requirements of broiler chickens, as recommended by the National Research Council [25]. All experimental diets were prepared in mash form. The basal diet was mixed first, and for the SBP-supplemented diets, the required amount of SBP was initially diluted with a small portion of the basal corn–soybean meal diet using a step-down mixing procedure to ensure even distribution. This premix was then thoroughly incorporated into the complete diet using a horizontal feed mixer. Antibiotics were incorporated with the same mixing procedure.

**Table 1:** Composition and nutrient levels of the experimental diets (starter and finisher phases).

Item	Starter (1–21 days)	Finisher (21–42 days)
Ingredient (%)		
Corn	54.9	58.7
Wheat bran	2.2	4.2
Calcium phosphate	1.1	1.6
Soybean meal	28.5	25.4
Fish meal	6.0	3.0
Limestone	1.2	1.2
DL-Methionine	0.3	0.1
Vitamin–mineral premix	0.5	0.5
Rapeseed oil	5.0	5.0
Sodium chloride	0.3	0.3
Calculated nutrient composition		
ME (kcal/kg)	3,000	3,100
CP (%)	20.54	19.76
Lysine (%)	1.19	1.10
Methionine + cystine (%)	0.93	0.75
Calcium (%)	1.01	0.94
Total phosphorus (%)	0.79	0.77
Available phosphorus (%)	0.45	0.40

CP = Crude protein, ME = Metabolizable energy. Vitamin–mineral premix supplied per kilogram of diet: Vitamin D3, 200 IU, Vitamin A, 1500 IU, Cobalamin, 0.01 mg, Biotin, 0.15 mg, Vitamin K3, 0.5 mg, Folic acid, 0.55 mg, Thiamine, 1.8 mg, Pyridoxine, 3.5 mg, Riboflavin, 3.6 mg, Vitamin E, 10 mg, D-pantothenic acid, 10 mg, Niacin, 35 mg, Selenium, 0.15 mg, Iodine, 0.35 mg, Copper, 8 mg, Zinc, 40 mg, Manganese, 60 mg, Iron, 80 mg. Diet nutrient levels were based on National Research Council recommendations [25].

After preparation, all diets were stored in double-layered opaque plastic bags at room temperature (approximately 20°C–25°C) in a dark, dry, and well-ventilated storage room. Fresh feed was prepared every two weeks to minimize nutrient degradation and to maintain the stability and antioxidant activity of SBP. The rapeseed oil included in the diet also helped protect fat-soluble components and reduce oxidative rancidity.

### Growth performance measurements

Initial body weight was recorded on day 1, with a coefficient of variation of 5%, indicating excellent uniformity among all treatment groups at the start of the experiment. Body weight and feed consumption per replicate cage were subsequently recorded on days 21 and 42. Average daily gain (ADG), average daily feed intake (ADFI), and feed conversion ratio (FCR) were calculated for the periods of days 1–21 and days 1–42. Data for ADG and FCR were corrected by adjusting the number of birds and total weight gain in replicate cages where mortality occurred during the respective period.

The following formulas were used for calculations:

ADG (g/bird/day) = (final body weight − initial body weight)/number of days

ADFI (g/bird/day) = (total feed offered − total feed residue)/(number of birds × number of days)

FCR (g/g) = total feed intake/total body weight gain

### Animal welfare and health monitoring

Broiler welfare was monitored throughout the 42-day experimental period. All birds were observed at least twice daily, in the morning and afternoon, by trained personnel for signs of distress, injury, or disease, including lethargy, inability to access feed or water, lameness, or severe respiratory symptoms. Birds showing severe and persistent pain or distress that could not be alleviated were humanely euthanized and recorded as mortalities.

Specific criteria for euthanasia included severe trauma, such as prolapse or non-ambulatory status, and advanced stages of systemic disease characterized by extreme lethargy and refusal to eat or drink for more than 24 h. All dead or removed birds were weighed, recorded, and the cause of mortality was documented when possible.

### Sample collection procedures

On days 21 and 42, blood and tissue samples were collected from 12 broilers per treatment group, with two birds randomly selected from each of the six replicate cages. All sampling was conducted in the morning between 8:00 and 10:00 a.m. to control for daily variation. Birds were humanely euthanized by cervical dislocation performed by trained personnel, in accordance with the guidelines of the Chinese Veterinary Medical Association.

To prevent cross-contamination, a fresh set of sterile instruments was used for each bird and for the collection of different tissues, and work surfaces were disinfected with 70% ethanol between birds. Blood samples were collected via wing vein puncture, and serum was separated by centrifugation at 1,000 × *g* for 10 min at 4°C, then stored at −20°C for up to two weeks until analysis. Following euthanasia, the intact intestine was removed from the abdominal cavity. For morphological analysis, duodenum, jejunum, and ileum samples were fixed in 10% neutral buffered formalin (Sigma-Aldrich, St. Louis, MO, USA) [26]. Additional duodenum and jejunum samples were snap-frozen in liquid nitrogen and stored at −80°C. Digestive enzyme activities were assessed using duodenal and cecal digesta samples, while antioxidant indices were measured in intestinal tissue from the middle portions of the duodenum and jejunum.

### Serum antioxidant index assay

Serum antioxidant indices, including glutathione peroxidase (GSH-Px), glutathione reductase (GR), superoxide dismutase (SOD), total antioxidant capacity (T-AOC), and malondialdehyde (MDA), were measured using commercial assay kits supplied by Nanjing Jiancheng Bioengineering Institute (Nanjing, China) [27]. The detection ranges were 10–1000 U/mL for GSH-Px, 1,000–10,000 U/mL for GR, 5–200 U/mL for SOD, 0.1–10 U/mL for T-AOC, and 0.5–50 nmol/mL for MDA. All assays were calibrated using standard curves provided by the manufacturer, and results were expressed as U/mL or nmol/mL of serum. All procedures were performed according to the manufacturer’s instructions.

### Intestinal antioxidant index analysis

Intestinal mucosal samples were homogenized in nine volumes (w/v) of ice-cold 0.1 M phosphate-buffered saline (PBS, pH 7.4) using a mechanical homogenizer. The homogenates were centrifuged at 10,000 × *g* for 10 min at 4°C with an Eppendorf 5430 R centrifuge, and the resulting supernatants were stored at −80°C. Protein concentrations were measured using a bicinchoninic acid (BCA) protein assay kit with bovine serum albumin as the standard. Levels of GSH-Px, GR, SOD, T-AOC, and MDA in intestinal homogenates were determined using commercial kits from Nanjing Jiancheng Bioengineering Institute. All values were expressed as U/mg protein or nmol/mg protein, following the manufacturer’s instructions.

### Measurement of sIgA

Jejunal mucosal sIgA levels were measured using a commercial chicken sIgA enzyme-linked immunosorbent assay kit (Catalog No. E02S002; China Institute of Atomic Energy, Beijing, China) and expressed as units per gram of protein [28]. Mucosal homogenates were diluted 1:10 with PBS before analysis. Samples were measured in duplicate with six biologically independent replicates per treatment group. Concentrations were calculated using a four-parameter logistic curve, normalized to protein concentration determined by BCA assay, and expressed as U/mg protein. All procedures followed the manufacturer’s instructions.

### Digestive enzyme activity assay

The activities of trypsin, chymotrypsin, lipase, and amylase in duodenal and cecal digesta samples were measured using commercial kits from Nanjing Jiancheng Bioengineering Institute (Nanjing, China). Casein, benzoyl-tyrosine ethyl ester, olive oil emulsion, and soluble starch served as substrates for trypsin, chymotrypsin, lipase, and amylase assays, respectively. Reactions were incubated at 37°C for 10 min (trypsin and chymotrypsin), 30 min (amylase), or 60 min (lipase). Enzyme activities were normalized to protein concentration, which was determined by BCA assay, and expressed as U/mg protein. All procedures followed the manufacturer’s instructions.

### Evaluation of intestinal morphology

Approximately 2 cm segments of the duodenum, jejunum, and ileum were collected and fixed in 10% neutral buffered formalin for 24–48 h. Tissues were dehydrated through graded ethanol solutions, cleared in xylene, and embedded in paraffin. Serial sections of 5 µm thickness were cut and stained with hematoxylin and eosin. Histomorphometric analysis was performed according to previously described methods of intestinal morphological measurements [21]. Villus height (VH) and crypt depth (CD) were measured using an Olympus IX81 microscope (Japan) and CellSens imaging software [29]. At least 10 intact and well-oriented villi and associated crypts were measured per intestinal segment per bird at 40× magnification, and the VH-to-CD ratio was calculated.

### Gene expression analysis by quantitative real-time polymerase chain reaction (qRT-PCR)

Total RNA was extracted using Thiocyanate–guanidinium isothiocyanate reagent, and RNA purity and concentration were measured spectrophotometrically, with A260/A280 ratios between 1.8 and 2.0. Genomic DNA contamination was eliminated with RNase-free DNase I. First-strand cDNA was synthesized from 1 µg of total RNA using the PrimeScript™ RT reagent kit with gDNA Eraser (TaKaRa, Japan). qRT-PCR was conducted using Power SYBR Green PCR Master Mix on a Bio-Rad MyiQ2 system (USA) with the following conditions: one cycle at 95°C for 5 min, followed by 40 cycles at 95°C for 15 s and 58°C for 45 s. β-actin served as the reference gene after confirming stable expression. Relative gene expression was calculated using the 2̅ΔΔCt method [30]. Primer sequences for CCL28, APRIL, and β-actin were synthesized by Sangon Biotechnology (Shanghai, China).

### Protein expression analysis by Western blot

Total protein was extracted from duodenal and jejunal tissues using RIPA buffer, and protein concentration was measured with a BCA assay kit. Equal amounts of protein (30 µg) were separated by 10% Sodium dodecyl sulfate–polyacrylamide gel electrophoresis and transferred onto polyvinylidene fluoride membranes. After blocking, the membranes were washed with TBS containing 0.5% Tween 20 and incubated overnight at 4°C with primary antibodies against TLR4 and β-actin. The membranes were then incubated with horseradish peroxidase-conjugated secondary antibodies and visualized using enhanced chemiluminescence. TLR4 band intensity was normalized to β-actin for quantitative analysis.

### Blinding procedures

A single-blind protocol was used for all laboratory analyses. Personnel performing biochemical assays, histological measurements, and molecular analyses were blinded to treatment assignment until data collection and statistical analysis were completed.

### Statistical analysis

All statistical analyses were conducted using SPSS Statistics version 21.0 (SPSS Inc., Chicago, IL, USA). The replicate cage served as the experimental unit for all performance variables, including ADG, ADFI, FCR, and mortality. For biochemical, immunological, histological, and molecular endpoints, birds were sampled as described (two birds per replicate cage). Values were analyzed using cage-based biological replicates (n = 6 per treatment) by averaging measurements within each replicate cage before performing statistical tests to prevent pseudo-replication. When laboratory assays were run in duplicate, the mean of the technical duplicates was used as the final value for each biological replicate.

Data were initially screened for completeness, outliers, and distributional properties. The assumptions of normality and homogeneity of variances were evaluated using the Shapiro–Wilk test and Levene’s test, respectively. When necessary, data were log10-transformed to meet model assumptions; if the transformation did not normalize residuals, non-parametric analyses were conducted (Kruskal–Wallis test followed by Dunn’s post hoc test with proper adjustment for multiple comparisons). Mortality (%) was compared among treatments using either chi-square or Fisher’s exact test, depending on expected cell counts.

For outcomes measured at a single time point, treatment effects were analyzed using one-way analysis of variance (ANOVA) under a CRD. The statistical model was:

Yij = μ + Ti + eij

where Yij is the observed response, μ is the overall mean, Ti is the fixed effect of dietary treatment (control, antibiotics, SBP-L, SBP-M, SBP-H), and eij is the residual error.

For variables measured at two sampling times (days 21 and 42), data were analyzed using a two-way ANOVA (treatment, time, and treatment × time interaction). When a significant interaction was detected, simple effects were examined by comparing treatments within each time point. For all ANOVA models, least-square means were compared using Duncan’s new multiple range test when the overall F-test was significant (*p* < 0.05). Results are presented as mean ± standard error (SE). Differences were considered statistically significant at P < 0.05, and 0.05 ≤ p < 0.10 was interpreted as a statistical trend where appropriate. Graphs were generated using GraphPad Prism version 8.00 (GraphPad Software Inc., San Diego, CA, USA).

## RESULTS

### Effects of dietary SBP on growth performance and mortality

**Figure 1:**
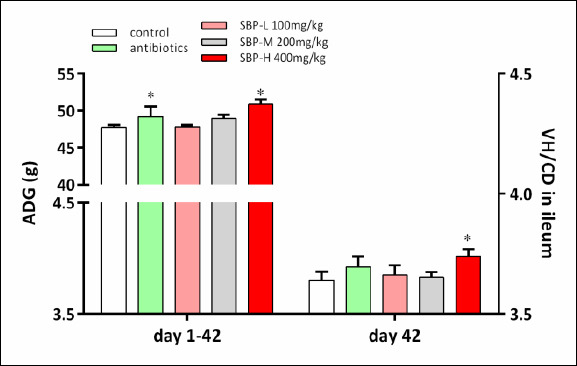
Effects of SBP supplementation on growth performance and intestinal morphology in broilers. SBP supplementation enhanced growth performance and intestinal morphology in broilers. ADG from day 1 to day 42 and VH/CD ratio in the ileum on day 42. Broilers received one of five diets: control, antibiotics, SBP-L, SBP-M, or SBP-H. Data were obtained from n = 6 biologically independent replicates (cages) per treatment. Statistical significance was determined by one-way analysis of variance followed by Duncan’s multiple range test. Asterisks indicate significant differences compared with the control group (*p < 0.05). The red arrow indicates an upward trend and dose-dependent response. ADG = Average daily gain, SBP = *Scutellaria baicalensis* polysaccharide, SEM = Standard error of the mean, VH/CD = Villus height-to-crypt depth ratio.

As shown in Table 2, dietary treatments significantly influenced the ADG of broilers during days 1–21 (p < 0.05). Broilers supplemented with SBP had higher ADG than the control group (p < 0.05). Over the entire experimental period (days 1–42), the ADG of broilers in the control and antibiotic groups was lower than that of broilers in the SBP-H group (p < 0.05; Figure 1). Notably, broilers fed 400 mg/kg SBP achieved a 3.4% higher ADG than those fed the antibiotic-supplemented diet (p < 0.05), indicating that growth performance in the SBP-H group was better than with in-feed antibiotics.

**Table 2:** Effects of dietary *SBP* supplementation on growth performance and mortality of broiler chickens.

Parameter	Control	Antibiotics	SBP-L	SBP-M	SBP-H	SEM	p-value
ADG (g)							
1–21 days	24.39^c^	24.72^bc^	25.96^a^	26.01^ab^	26.12^b^	0.177	0.003
22–42 days	70.97	73.32	71.32	73.01	75.59	0.843	0.063
1–42 days	47.74^b^	49.17^ab^	47.79^b^	48.96^b^	50.88^a^	0.481	0.043
ADFI (g)							
1–21 days	42.26	42.38	42.03	41.81	42.45	0.168	0.628
22–42 days	138.38	141.29	138.49	136.07	145.01	0.601	0.223
1–42 days	88.91	90.29	88.77	89.98	92.03	0.912	0.174
FCR (g/g)							
1–21 days	1.71^a^	1.70^a^	1.69^ab^	1.65^b^	1.64^b^	0.014	0.001
22–42 days	1.95	1.92	1.93	1.94	1.91	0.019	0.589
1–42 days	1.85	1.83	1.84	1.83	1.80	0.011	0.074
Mortality (%)	2.38	1.49	2.44	2.06	1.66	0.42	0.092

ADG = Average daily gain, ADFI = Average daily feed intake, FCR = Feed conversion ratio, SBP = *Scutellaria baicalensis* polysaccharide, SEM = Standard error of the mean. Control = Antibiotic-free basal diet, Antibiotics = Basal diet supplemented with colistin sulfate and virginiamycin, SBP-L = 100 mg *S. baicalensis* polysaccharide/kg feed, SBP-M = 200 mg S. baicalensis polysaccharide/kg feed, SBP-H = 400 mg *S. baicalensis* polysaccharide/kg feed. (a–c) Means within a row with different superscripts differ significantly (p < 0.05).

ADFI did not significantly differ among dietary treatments during either growth phase. However, FCR was significantly lower in SBP-supplemented groups compared to the control and antibiotic groups during days 1–21 (p < 0.05), indicating improved feed efficiency during the starter period.

Mortality was monitored daily throughout the experiment. The overall mortality rate was low at 3% and did not differ significantly among dietary treatments (p > 0.05; Table 2). These findings suggest that dietary SBP supplementation did not have any negative effects on broiler survival under the conditions of this study.

## Effects of dietary SBP on serum antioxidant status

**Figure 2:**
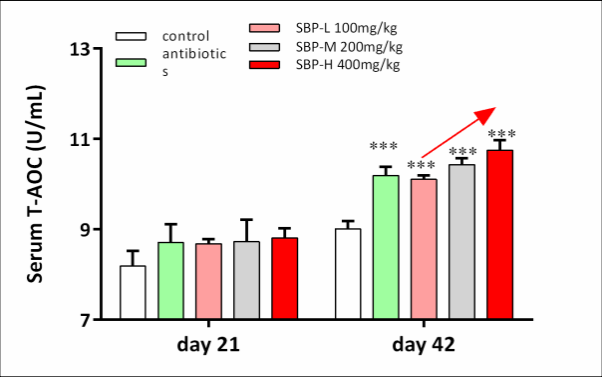
Effects of SBP supplementation on systemic antioxidant capacity and digestive enzyme activity in broilers. SBP supplementation enhanced systemic antioxidant capacity and digestive enzyme activity in broilers. Serum T-AOC on day 21 and 42. Broilers received one of five diets: control, antibiotics, SBP-L, SBP-M, or SBP-H. Data points represent mean ± SEM of n = 6 replicate cages per treatment group. Statistical significance was determined by one-way analysis of variance followed by Duncan’s multiple range test. Asterisks indicate significant differences compared with the control group (*p < 0.05, **p < 0.01, ***p < 0.001). The red arrow indicates an upward trend and dose-dependent response. SBP = *Scutellaria baicalensis* polysaccharide, SEM = Standard error of the mean, T-AOC = Total antioxidant capacity.

Serum antioxidant parameters are shown in Table 3 and Figure 2A. On day 21, serum levels of GSH-Px and SOD were significantly higher in the antibiotic and SBP-supplemented groups than in the control group (p < 0.05). Dietary SBP supplementation also significantly increased serum GR activity compared to the control group (p < 0.05). Conversely, serum MDA levels were significantly lower in both the antibiotic and SBP groups compared to the control group (p < 0.05), with no significant difference between the SBP and antibiotic treatments (p > 0.05).

**Table 3:** Effect of dietary SBP supplementation on serum antioxidant indices in broiler chickens.

Parameter	Control	Antibiotics	SBP-L	SBP-M	SBP-H	SEM	p-value
21 days							
GSH-Px (U/mL)	791.3_c_	813.2^b^	822.4^a^	829.1^a^	840.4^a^	5.08	0.001
GR (U/mL)	4290^b^	4360^b^	4810^a^	4870^a^	4910^a^	85.03	0.010
SOD (U/mL)	70.88^b^	75.11^a^	75.01^a^	76.13^a^	77.44^a^	0.661	0.003
T-AOC (U/mL)	8.19	8.71	8.68	8.73	8.81	0.071	0.087
MDA (nmol/mL)	4.93^a^	4.21^b^	4.41^b^	4.37^b^	4.16^b^	0.109	0.033
42 days							
GSH-Px (U/mL)	984.9^b^	1108.3^a^	1089.4^a^	1101.9^a^	1163.7^a^	12.8	0.012
GR (U/mL)	5010^b^	5240^b^	5610^a^	5770^a^	5930^a^	74.04	0.001
SOD (U/mL)	84.88^b^	89.01^ab^	89.43^a^	90.72^a^	91.33^a^	1.002	0.037
T-AOC (U/mL)	9.01^b^	10.19^a^	10.11^a^	10.43^a^	10.75^a^	0.178	0.007
MDA (nmol/mL)	4.61	4.58	4.33	4.27	4.19	0.198	0.762

GSH-Px = Glutathione peroxidase, GR = Glutathione reductase, SOD = Superoxide dismutase, T-AOC = Total antioxidant capacity, MDA = Malondialdehyde, SEM = Standard error of the mean. Control = Antibiotic-free basal diet, Antibiotics = Basal diet supplemented with colistin sulfate and virginiamycin, SBP-L = 100 mg S. baicalensis polysaccharide/kg feed, SBP-M = 200 mg S. baicalensis polysaccharide/kg feed, SBP-H = 400 mg *S. baicalensis* polysaccharide/kg feed. (a–c) Means within a row with different superscripts differ significantly (p < 0.05).

On day 42, serum MDA levels did not differ among dietary treatments (p > 0.05). However, serum SOD and GR activities were significantly higher in the SBP-supplemented groups than in the control group (p < 0.05). Additionally, serum GSH-Px activity and T-AOC were significantly increased in both the antibiotic and SBP groups compared to the control group (p < 0.05).

### Intestinal mucosal antioxidant capacity and immune responses

Dietary SBP supplementation improved intestinal antioxidant function in a segment- and time-dependent way. In the duodenum, SOD activity was notably increased on day 21 after SBP supplementation (p < 0.05; Table 4). By day 42, the activities of GSH-Px, T-AOC, and GR in the duodenum were significantly higher in the antibiotic and SBP groups compared to the control group (p < 0.05).

**Table 4:** Effect of dietary SBP supplementation on antioxidant indices in the duodenum of broiler chickens.

Parameter	Control	Antibiotics	SBP-L	SBP-M	SBP-H	SEM	p-value
21 days							
GSH-Px (U/mg prot)	92.9	95.01	96.13	99.05	100.9	1.279	0.111
GR (U/mg prot)	2770	3140	3400	3560	3710	130	0.183
SOD (U/mg prot)	50.98^b^	53.71^ab^	53.68^ab^	55.46^a^	56.97^a^	0.710	0.041
T-AOC (U/mg prot)	3.87	5.01	4.89	5.01	5.14	0.313	0.069
MDA (nmol/mg prot)	3.32	3.19	3.03	2.89	2.66	0.154	0.449
42 days							
GSH-Px (U/mg prot)	76.01^b^	87.13^a^	88.43^a^	90.19^a^	93.03^a^	1.774	0.001
GR (U/mg prot)	2980^b^	5310^a^	4990^a^	5180^a^	5440^a^	391	0.012
SOD (U/mg prot)	83.81	87.60	86.72	89.93	90.44	1.839	0.136
T-AOC (U/mg prot)	7.08^b^	8.52^a^	8.33^a^	8.40^a^	8.51^a^	0.188	0.033
MDA (nmol/mg prot)	5.91	5.46	5.01	4.78	4.69	0.146	0.086

GSH-Px = Glutathione peroxidase, GR = Glutathione reductase, SOD = Superoxide dismutase, T-AOC = Total antioxidant capacity, MDA = Malondialdehyde, SEM = Standard error of the mean. U/mg prot = Units per milligram protein. Control = Antibiotic-free basal diet, Antibiotics = Basal diet supplemented with colistin sulfate and virginiamycin, SBP-L = 100 mg *S. baicalensis* polysaccharide/kg feed, SBP-M = 200 mg *S. baicalensis* polysaccharide/kg feed, SBP-H = 400 mg *S. baicalensis* polysaccharide/kg feed. (a–c) Means within a row with different superscripts differ significantly (p < 0.05).

In the jejunum, dietary SBP supplementation significantly increased GSH-Px and T-AOC activities on day 21 (p < 0.05; Table 5). On day 42, GSH-Px and SOD activities in the jejunum were significantly higher in both the antibiotic and SBP groups compared with the control group (p < 0.05), while jejunal MDA concentrations were significantly lower (p < 0.05).

**Table 5:** Effect of dietary SBP supplementation on antioxidant indices in the jejunum of broiler chickens.

Parameter	Control	Antibiotics	SBP-L	SBP-M	SBP-H	SEM	p-value
21 days							
GSH-Px (U/mg prot)	96.13_c_	101.4^bc^	104.1^ab^	110.9^a^	118.7^a^	1.367	0.001
GR (U/mg prot)	2440	2610	2790	2910	3040	144	0.281
SOD (U/mg prot)	50.04	51.33	53.01	53.44	55.71	1.098	0.218
T-AOC (U/mg prot)	4.79^b^	5.61^a^	5.51^a^	5.53^a^	5.57^a^	0.023	0.041
MDA (nmol/mg prot)	2.88	2.64	2.58	2.48	2.39	0.091	0.217
42 days							
GSH-Px (U/mg prot)	102.1^b^	123.0^a^	117.9^a^	119.2^a^	121.0^a^	2.08	0.001
GR (U/mg prot)	4200	5640	5610	5470	5290	241	0.076
SOD (U/mg prot)	81.08^b^	92.44^a^	91.78^a^	92.30^a^	94.01^a^	1.571	0.013
T-AOC (U/mg prot)	7.89	8.64	8.71	8.81	8.98	0.149	0.089
MDA (nmol/mg prot)	5.34^a^	4.11^b^	4.61^b^	4.47^b^	4.39^b^	0.081	0.031

GSH-Px = Glutathione peroxidase, GR = Glutathione reductase, SOD = Superoxide dismutase, T-AOC = Total antioxidant capacity, MDA = Malondialdehyde, SEM = Standard error of the mean. U/mg prot = Units per milligram protein. Control = Antibiotic-free basal diet, Antibiotics = Basal diet supplemented with colistin sulfate and virginiamycin, SBP-L = 100 mg *S. baicalensis* polysaccharide/kg feed, SBP-M = 200 mg *S. baicalensis* polysaccharide/kg feed, SBP-H = 400 mg *S. baicalensis* polysaccharide/kg feed. (a–c) Means within a row with different superscripts differ significantly (p < 0.05).

**Figure 3:**
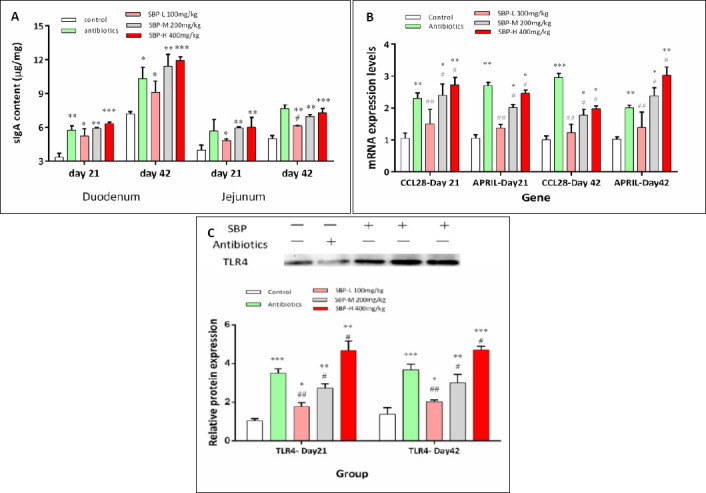
Effects of SBP supplementation on intestinal immune parameters in broilers. SBP supplementation modulated intestinal immune responses in broilers. (A) sIgA content in the duodenum and jejunum on days 21 and 42. (B) Relative mRNA expression of CCL28 and APRIL in the jejunal mucosa on days 21 and 42. (C) Representative western blot images and quantitative analysis of TLR4 protein expression in the jejunum on days 21 and 42.

Regarding mucosal immunity, sIgA levels in the duodenum and jejunum were significantly higher in the SBP-supplemented groups compared to the control group on both days 21 and 42 (p < 0.05; Figure 3A). Additionally, the mRNA levels of CCL28 and APRIL were significantly increased in the SBP-M and SBP-H groups at both sampling points (p < 0.05; Figure 3B). Consistent with these results, TLR4 protein levels were also significantly higher in the SBP-M and SBP-H groups on days 21 and 42 (p < 0.05; Figure 3C).

Broilers received one of five diets: control, antibiotics, SBP-L, SBP-M, or SBP-H. Data were obtained from n = 6 biologically independent replicates (cages) per treatment. Panels A and B show mean ± SEM. In panel C, quantitative values are presented as mean ± SEM (n = 6), normalized to β-actin and expressed relative to the control group. Original western blot images are shown above the corresponding graphs, with each lane representing one biological replicate. Statistical analysis was performed using one-way analysis of variance followed by Duncan’s multiple range test. Asterisks indicate significant differences compared with the control group (*p < 0.05, **p < 0.01, ***p < 0.001), and hash symbols indicate significant differences compared with the antibiotics group (#p < 0.05, ##p < 0.01, ###p < 0.001). APRIL = A proliferation-inducing ligand, CCL28 = C-C motif chemokine ligand 28, sIgA = Secretory immunoglobulin A, SBP = *Scutellaria baicalensis* polysaccharide, SBP-L = 100 mg/kg SBP, SBP-M = 200 mg/kg SBP, SBP-H = 400 mg/kg SBP, SEM = Standard error of the mean, TLR4 = Toll-like receptor 4.

### Digestive enzyme activity and intestinal morphology

**Figure 4:**
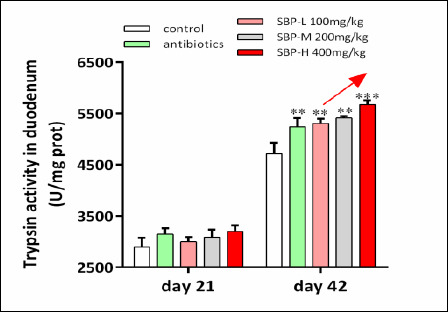
Trypsin activity in the duodenum on day 42. Broilers received one of five diets: control, antibiotics, SBP-L, SBP-M, or SBP-H. Data points represent mean ± SEM of n = 6 replicate cages per treatment group. Statistical significance was determined by one-way analysis of variance followed by Duncan’s multiple range test. Asterisks indicate significant differences compared with the control group (*p < 0.05, **p < 0.01, ***p < 0.001). The red arrow indicates an upward trend and dose-dependent response. SBP = *Scutellaria baicalensis* polysaccharide, SEM = Standard error of the mean.

Digestive enzyme activities are summarized in Table 6 and Figure 4. On day 21, duodenal lipase activity was significantly higher in the antibiotic group compared to the control group (p < 0.05). On day 42, trypsin activity was significantly increased in SBP-supplemented groups (p < 0.05). Additionally, chymotrypsin, lipase, and amylase activities were significantly higher in both the antibiotic and SBP groups than in the control group (p < 0.05).

**Table 6:** Effect of dietary SBP supplementation on digestive enzyme activities in the duodenum of broiler chickens.

Parameter	Control	Antibiotics	SBP-L	SBP-M	SBP-H	SEM	p-value
21 days							
Trypsin (U/mg)	2893	3144	2997	3086	3179	46	0.096
Chymotrypsin (U/mg protease)	3.79	4.20	4.13	4.39	4.56	0.089	0.054
Lipase (U/g prot)	33.78^b^	41.03^a^	37.46^ab^	38.01^ab^	38.49^ab^	0.611	0.032
Amylase (U/mg prot)	3.71	4.60	4.49	4.53	4.67	0.161	0.107
42 days							
Trypsin (U/mg)	4712.2^b^	5239.0^ab^	5307.0^a^	5411.0^a^	5673.0^a^	123.1	0.008
Chymotrypsin (U/mg protease)	6.98^b^	8.91^a^	8.58^a^	8.69^a^	8.84^a^	0.109	0.001
Lipase (U/g prot)	96.81^b^	117.3^a^	104.0^a^	112.7^a^	119.6^a^	2.01	0.003
Amylase (U/mg prot)	5.44^b^	5.91^a^	5.88^a^	6.01^a^	6.14^a^	0.067	0.007

SEM = Standard error of the mean. U/mg prot = Units per milligram protein, U/g prot = Units per gram protein. Control = Antibiotic-free basal diet, Antibiotics = Basal diet supplemented with colistin sulfate and virginiamycin, SBP-L = 100 mg *S. baicalensis* polysaccharide/kg feed, SBP-M = 200 mg *S. baicalensis* polysaccharide/kg feed, SBP-H = 400 mg *S. baicalensis* polysaccharide/kg feed. (a, b) Means within a row with different superscripts differ significantly (p < 0.05).

Intestinal morphology data are shown in Table 7 and Figure 1. On day 21, there were no significant differences in VH or CD in the duodenum, jejunum, or ileum among dietary treatments (p > 0.05). However, the VH-to-CD (VH/CD) ratio in the duodenum, jejunum, and ileum was significantly higher in the SBP-H group compared with the control group (p < 0.05).

**Table 7:** Effect of dietary SBP supplementation on the intestinal morphology of broiler chickens.

Parameter	Control	Antibiotics	SBP-L	SBP-M	SBP-H	SEM	p-value
Duodenum							
VH (μm) d21	1806	2014	1908	2018	2115	78.76	0.078
CD (μm) d21	289	319	295	308	319	9.81	0.593
VH/CD d21	6.25^b^	6.31^b^	6.47^b^	6.55^b^	6.63^a^	0.073	0.038
VH (μm) d42	1901^b^	2146^b^	2177^b^	2203^a^	2247^a^	48.6	0.044
CD (μm) d42	299^b^	289^b^	267^a^	249^a^	231_c_	2.73	0.025
VH/CD d42	6.36_c_	7.43^a^	8.15^a^	8.85^a^	9.74^ab^	0.19	0.017
Jejunum							
VH (μm) d21	1135	1304	1288	1331	1357	178.9	0.417
CD (μm) d21	218	209	208	211	203	7.41	0.804
VH/CD d21	5.21_c_	6.23^a^	6.19^a^	6.31^a^	6.68^ab^	0.092	0.006
VH (μm) d42	1283	1366	1318	1407	1458	41.3	0.668
CD (μm) d42	218	195	188	190	187	5.77	0.922
VH/CD d42	5.88_c_	7.01^a^	7.01^a^	7.29^a^	7.80^ab^	0.11	0.026
Ileum							
VH (μm) d21	669	728	657	681	690	39.6	0.704
CD (μm) d21	199	192	182	189	177	3.08	0.343
VH/CD d21	3.36^b^	3.79^a^	3.61^a^	3.60^a^	3.90^a^	0.058	0.033
VH (μm) d42	691	749	704	709	716	22.3	0.591
CD (μm) d42	182	191	183	185	178	4.01	0.815
VH/CD d42	3.80^b^	3.92^b^	3.85^b^	3.83^b^	4.02^a^	0.061	0.047

VH = Villus height, CD = Crypt depth, VH/CD = Villus height-to-crypt depth ratio, SEM = Standard error of the mean. Control = Antibiotic-free basal diet, Antibiotics = Basal diet supplemented with colistin sulfate and virginiamycin, SBP-L = 100 mg *S. baicalensis* polysaccharide/kg feed, SBP-M = 200 mg *S. baicalensis* polysaccharide/kg feed, SBP-H = 400 mg *S. baicalensis* polysaccharide/kg feed. (a–c) Means within a row with different superscripts differ significantly (p < 0.05).

On day 42, duodenal VH was significantly increased in the antibiotic, SBP-M, and SBP-H groups (p < 0.05), while duodenal CD was significantly reduced following SBP supplementation (p < 0.05). VH and CD in the jejunum and ileum were not significantly affected by dietary treatments (p > 0.05). However, the VH/CD ratio in the duodenum and jejunum was significantly increased with SBP supplementation (p < 0.05). A significant increase in ileal VH/CD ratio was observed only in the SBP-H group (p < 0.05).

## DISCUSSION

### Effects of SBP on growth performance and dose-dependent responses

This study shows that dietary supplementation with *S. baicalensis* polysaccharide (SBP) benefits broiler growth, antioxidant levels, immune health, digestion, and gut health. The improvements in ADG and FCR match previous findings that highlight the effectiveness of herbal extracts and polysaccharides in boosting animal production performance [31, 32]. The optimal SBP dosage range found in this study (200–400 mg/kg) is consistent with effective doses of other well-known TCM–derived polysaccharides [32, 33], further supporting their use as functional feed additives.

Notably, SBP served as an effective alternative to in-feed antibiotics, as broilers in the SBP-H group showed better ADG over the entire 42-day period compared to the antibiotic group, supporting earlier findings on the growth-promoting effects of herbal additives [33–35]. Clear dose-dependent responses were seen, especially in systemic antioxidant capacity, demonstrated by increased serum T-AOC, and in the upregulation of immune-related genes APRIL and CCL28. The better performance of the SBP-H (400 mg/kg) group indicates that higher SBP inclusion levels might be necessary to fully activate complex systemic immunomodulatory pathways, such as the proposed TLR4–sIgA axis, while more localized intestinal effects, including digestive enzyme activity and antioxidant enzyme expression, were already significantly improved at the medium dose (200 mg/kg).

### Enhancement of systemic and intestinal antioxidant capacity

The improved antioxidant status seen in the serum, duodenum, and jejunum of SBP-fed broilers, marked by increased activities of GSH-Px, SOD, GR, and T-AOC and lower MDA levels, shows that SBP provides strong protective effects against oxidative stress [36]. Although serum antioxidant measurements are often reported, this study offers a more thorough assessment by evaluating the intestinal mucosal redox environment, which has received less attention.

Significant improvements in duodenal and jejunal antioxidant parameters emphasize the local action of SBP within the gastrointestinal tract, which is essential for maintaining gut integrity and physiological function. The well-known antioxidant activity of *S. baicalensis* [37], along with the observed increase in endogenous antioxidant enzymes, indicates an activated cellular defense system [36, 38]. Additionally, the ability of herbal extracts to inhibit lipid peroxidation has been consistently documented in earlier studies [39], further supporting the antioxidative role of SBP observed in this work.

### Modulation of intestinal mucosal immunity through the TLR4–sIgA axis

The beneficial effects of SBP on intestinal health, especially the notable increase in sIgA levels, may be closely connected to the metabolic pathway of this polysaccharide. sIgA is key to mucosal immunity, helping prevent pathogen adherence and maintain intestinal balance [40]. SBP is a high-molecular-weight polysaccharide that mostly resists degradation in the upper gastrointestinal tract, with over 80% reaching the colon intact, where it may engage immune pathways, such as TLR4 signaling [41].

Based on the current findings, a sequential mechanistic model can be suggested. First, dietary SBP largely bypasses digestion in the upper gastrointestinal tract and reaches the colon intact [41], where it may act directly as a microbe-associated molecular pattern recognized by PRRs, including TLR4, on immune cells, or more plausibly, serve as a fermentable substrate for the gut microbiota. Second, microbiota-derived metabolites, such as short-chain fatty acids (SCFAs), or shifts in microbial composition may increase TLR4 expression in the intestinal mucosa [42]. Third, activation of the TLR4 pathway in antigen-presenting cells, including dendritic cells, can enhance cytokine and co-stimulatory molecule production, thereby promoting B-cell class switching toward IgA production [43]. This mechanistic framework aligns with the observed upregulation of APRIL and CCL28, which are crucial for B-cell recruitment and the formation of IgA-secreting plasma cells [44].

While these findings differ from reports describing TLR4 inhibition by Astragalus polysaccharides [44], they align with studies showing increased TLR4 expression after supplementation with *Caulis spatholobi* poly-saccharides [45], indicating that immunomodulatory effects may be specific to certain polysaccharides. The well-established connection between gut microbiota and the immune system [43], along with evidence from *in ovo* polysaccharide administration studies demonstrating enhanced sIgA production [46], further supports the immunoregulatory role of SBP. Overall, the systemic and mucosal improvements observed in this study support the concept of the gut–immune axis, in which localized luminal interactions can coordinate local and systemic immune responses without requiring systemic absorption of the polysaccharide itself.

### Role of SBP phytochemical composition and microbiota modulation

The superior growth performance and health benefits observed in SBP-fed broilers may not solely reflect the general properties of polysaccharides but may also be linked to the unique phytochemical characteristics of SBP. Unlike many other herbal polysaccharides, SBP contains distinctive structural features, including flavonoid-linked polysaccharide complexes and arabinogalactans, which exhibit particularly strong antioxidant activities [37]. This unique biochemical profile likely contributes to the robust, multi-systemic effects observed, positioning SBP as a promising feed additive with a well-defined chemical basis for its biological activity.

Beyond direct immune interactions, SBP may influence its beneficial effects by modulating the gut microbiota. Many plant-derived polysaccharides act as prebiotics [45], selectively encouraging the growth of beneficial microbes that produce metabolites such as SCFAs. These metabolites improve intestinal barrier function, reduce inflammation, and directly promote sIgA secretion [46]. Therefore, the increased levels of TLR4 and sIgA observed in this study may be an indirect consequence of SBP-driven changes in the microbiota. To confirm this idea, future studies using 16S rRNA gene sequencing or metagenomic techniques are needed.

It is also important to note that the biological activity of the SBP extract may not be solely due to the polysaccharide fraction. *S. baicalensis* contains bioactive flavonoids, including baicalin and baicalein, which are well-known for their antioxidant and anti-inflammatory properties [47]. Although the extract was mainly characterized as a polysaccharide, trace co-extracted flavonoids may have exerted synergistic effects, contributing to the overall health benefits observed in the broilers.

### Improvement of digestive enzyme activity and intestinal morphology

The enhanced activity of digestive enzymes, including trypsin, chymotrypsin, lipase, and amylase, especially in the duodenum on day 42, indicates that SBP promotes enzyme secretion or activity, thereby enhancing nutrient digestion and absorption. These functional improvements are closely linked to the observed gains in intestinal morphology. Increases in VH and the VH-to-CD (VH/CD) ratio in the duodenum and jejunum are particularly important, as a higher VH/CD ratio increases the absorptive surface area and boosts digestive efficiency [47, 48].

The morphological improvements observed in this study align with findings from research on other herbal extracts [49, 50], Astragalus polysaccharides [20], and the combined use of essential oils and organic acids [51]. CD is directly related to epithelial cell proliferation and villus renewal [52], and a decrease in CD may indicate reduced epithelial turnover associated with lower inflammatory stress. Supporting this interpretation, Nabati *et al*. [53] reported a reduction in duodenal CD in broilers, attributed to less epithelial damage and atrophy resulting from decreased exposure to antigenic proteins and lectins. Given the established role of antioxidant activity in maintaining intestinal structure [54], the antioxidant properties of SBP are likely to support the observed improvements in intestinal architecture, thereby enhancing nutrient absorption and growth performance.

## CONCLUSION

This study shows that supplementing diets with *S. baicalensis* polysaccharide (SBP) significantly improves broiler growth, antioxidant defenses, digestion, intestinal structure, and mucosal immunity. Broilers given SBP, especially at 400 mg/kg, had better ADG and feed efficiency compared to both the antibiotic-free control and the antibiotic group. SBP significantly boosted systemic and intestinal antioxidant enzymes (GSH-Px, SOD, GR, T-AOC) while lowering lipid peroxidation, indicating strong protection against oxidative stress. Additionally, SBP increased digestive enzyme activity, promoted taller villi and higher villus-to-crypt ratios, and elevated immune markers such as sIgA, APRIL, CCL28, and TLR4, collectively suggesting improved intestinal health and immune function.

From a practical perspective, SBP at inclusion levels of 200–400 mg/kg was both effective and safe, with the higher dose (400 mg/kg) consistently delivering the most significant benefits. These results suggest that SBP can be a practical natural alternative to antibiotic growth promoters in broiler production, especially under post-antibiotic regulatory conditions. The improvements seen in growth efficiency, gut health, and antioxidant status are directly relevant to commercial poultry operations, as they could lead to better productivity, lower disease risk, and increased resilience to environmental and physiological stressors.

A key strength of this study is its integrated, multi-dimensional approach. Unlike studies that focus on isolated performance or immune parameters, this work simultaneously assesses growth performance, systemic and intestinal antioxidant capacity, digestive enzyme activity, intestinal morphology, and mucosal immune signaling. This comprehensive evaluation offers strong mechanistic insight into how SBP exerts its beneficial effects and supports its classification as a multifunctional feed additive rather than a single-target growth promoter.

Despite these strengths, some limitations should be recognized. The SBP used was a commercial extract, and although the purity and extraction method were disclosed, detailed structural analysis (e.g., molecular weight distribution, monosaccharide composition, and glycosidic linkages) was not conducted. Additionally, the gut microbiota was not directly examined, which limits the ability to definitively connect SBP supplementation to microbiome-mediated mechanisms. The evaluation of systemic health was also limited to antioxidant indices, without considering broader hematological or immunological markers.

Future studies should focus on clarifying the structure–activity relationship of SBP through advanced chemical analysis and directly examining gut microbiota modulation using *16S rRNA* gene sequencing or metagenomic methods. Long-term feeding trials, pathogen challenge tests, and thorough safety and toxicity assessments are necessary to confirm the sustainability and reliability of SBP effects. Additionally, economic analyses evaluating cost–benefit ratios in commercial settings will be essential for supporting large-scale adoption.

In conclusion, dietary *S. baicalensis* polysaccharide offers a scientifically supported, practical, and sustainable alternative to antibiotic growth promoters in broiler production. By simultaneously enhancing growth performance, antioxidant capacity, digestive efficiency, intestinal structure, and mucosal immunity, SBP promotes overall broiler health and productivity. These findings provide strong evidence for incorporating SBP into antibiotic-free poultry feeding strategies and offer valuable insight into developing resilient and sustainable poultry production systems.

## DATA AVAILABILITY

The supplementary data can be made available from the corresponding author upon request.

## AUTHORS’ CONTRIBUTIONS

YZ and JZ: Experimental design and drafted and edited the manuscript. SL: Performed the experiments, analyzed the data, and drafted the manuscript. QZ, YW, WZ, and LL: Performed the experiments and analyzed the data. All authors have read and approved the final version of the manuscript.
